# Paracoccidioidomycosis as an opportunistic manifestation of acquired immunodeficiency syndrome

**DOI:** 10.1590/0037-8682-0536-2021

**Published:** 2022-02-25

**Authors:** Paulo Ricardo Gomes dos Santos, Marilda Aparecida Milanez Morgado de Abreu

**Affiliations:** 1 Universidade do Oeste Paulista, Programa de Pós-Graduação Lato Sensu em Dermatologia, Presidente Prudente, SP, Brasil.

Paracoccidioidomycosis is a systemic mycosis caused by fungi of the genus *Paracoccidioides* (*P. brasiliensis* and *P. lutzi*), endemic in tropical and subtropical areas of Latin America and more prevalent in Brazil^1^. Co-infection with human immunodeficiency virus (HIV) and consequent immunosuppression may hinder its diagnosis because there may be an overlap of the acute/subacute and chronic forms in the same individual^2^ ^,^ ^3^.

**FIGURE 1:**
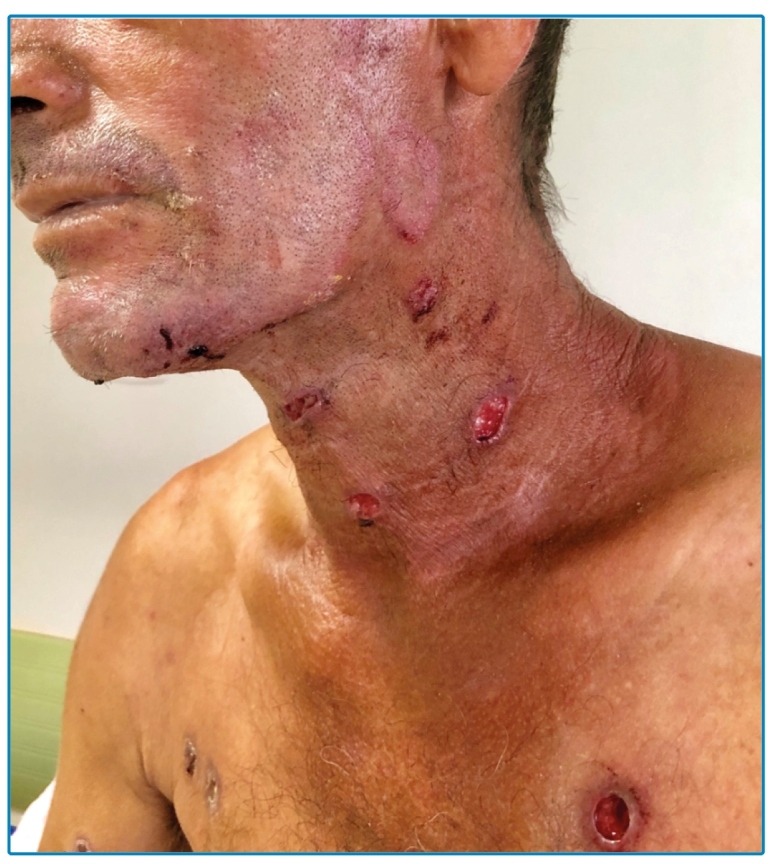
Ulcerated lesions with raised edges and granular bottom in the anterior cervical and thorax regions.

**FIGURE 2:**
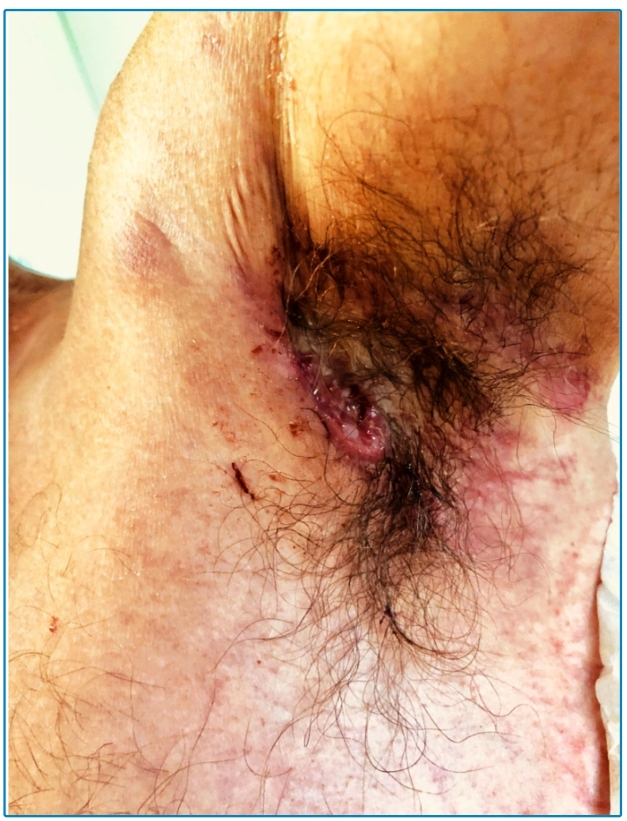
Ulcerated lesion with drainage of purulent secretion in the left axillary region.

**FIGURE 3:**
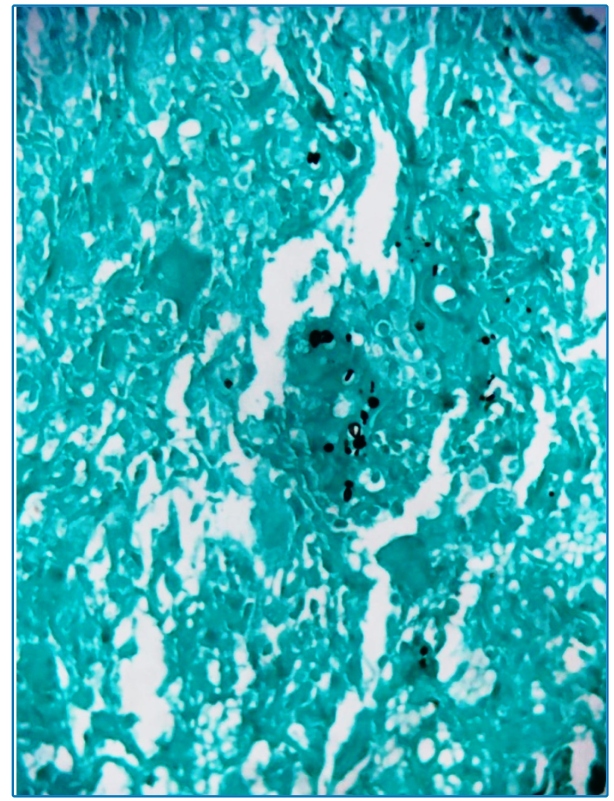
800x (400x + 2x Zoom) - Grocott - Fungal spores with varied sizes and multiple budding, compatible with *Paracoccidioides*.

A 45-year-old male patient living on the street sought care for tegumental lesions without improvement by antibiotic therapies that he had been taking for five months. He presented erythematous papules on the trunk, slightly painful ulcers with well-delimited and slightly elevated edges, and a granular and bleeding background in the anterior cervical area, face, nape, upper limbs, and thorax (Figure 1), and purulent secretion in the left armpit (Figure 2). His clinical and histopathological diagnoses were paracoccidioidomycosis ( Figure 3). Based on the laboratory investigation, the patient was diagnosed with AIDS (CD4 52 cel/mcL lymphocytes).

As an opportunistic disease, paracoccidioidomycosis occurs in individuals with advanced immunodeficiency, suggesting exuberant lymphatic involvement, fistulization, polymorphic skin lesions, and severe and disseminated symptoms, and it has high morbidity and mortality^2^ ^,^ ^3^.

Endemic systemic mycoses have increased in frequency in recent years in immunocompromised patients^1^ ^,^ ^2^ ^,^ ^3^. Thus, skin lesions indicative of paracoccidioidomycosis, especially those with severe skin and lymphatic system involvement, should alert the clinician not only to the possibility of systemic infection, prompting careful evaluation of the main organs targeted by mycosis, but also to the possibility of co-infection with HIV.
